# Hemodynamics of cerebral bridging veins connecting the superior sagittal sinus based on numerical simulation

**DOI:** 10.1186/s12938-018-0466-8

**Published:** 2018-03-20

**Authors:** Youyu Zhu, Feng Wang, Xuefei Deng

**Affiliations:** Department of Anatomy, Anhui Medical University, 81 Meishan Road, Hefei, 230032 China

**Keywords:** Cerebral bridging veins, Cerebral venous thrombosis, Wall shear stress, Computational fluid dynamics

## Abstract

**Background:**

The physiological and hemodynamic features of bridging veins involve wall shear stress (WSS) of the cerebral venous system. Based on the data of cadavers and computational fluid dynamics software pack, the hemodynamic physical models of bridging veins (BVs) connecting superior sagittal sinus (SSS) were established.

**Results:**

A total of 137 BVs formed two clusters along the SSS: anterior group and posterior group. The diameters of the BVs in posterior group were larger than of the anterior group, and the entry angle was smaller. When the diameter of a BV was greater than 1.2 mm, the WSS decreased in the downstream wall of SSS with entry angle less than 105°, and the WSS also decreased in the upstream wall of BVs with entry angle less than 65°. The minimum WSS in BVs was only 63% of that in SSS. Compared with the BVs in anterior group, the minimum WSS in the posterior group was smaller, and the distance from location of the minimum WSS to the dural entrance was longer.

**Conclusion:**

The cerebral venous thrombosis occurs more easily when the diameter of a BV is greater than 1.2 mm and the entry angle is less than 65°. The embolus maybe form earlier in the upstream wall of BVs in the posterior part of SSS.

**Electronic supplementary material:**

The online version of this article (10.1186/s12938-018-0466-8) contains supplementary material, which is available to authorized users.

## Background

Compared with the cerebral artery system, the cerebral venous system is usually asymmetric and its variability is greater, which makes it prone to venous thrombus and a variety of neurological disorders. With the development of medical imaging technology, especially with the rapid development of magnetic resonance technology [1–3], the diseases related to the cerebral venous system are more generally known and valued by clinics. This has prompted research into the hemodynamics of the cerebral venous system. Cerebral venous thrombosis is one of the most common of cerebral venous diseases [4]. The patients often develop symptoms of intracranial hemorrhage, cerebral edema, venous infarction and even death because of not getting timely treatment [5]. In clinical cure cases, there are a considerable number of patients with varying degrees of sequelae [5, 6] and significantly decreased quality of life. This phenomenon is largely due to not having timely diagnosis, and may delay the best treatment time.

The direct or indirect signs of thrombosis in radiographic images are an important basis for the diagnosis of cerebral venous thrombosis [7, 8]. Early clinical symptoms of most patients with thrombosis are atypical. There is no obvious manifestation of venous reflux obstruction. The restriction of imaging technology and the difficulty in determining the location of thrombus has led to difficulty in the early diagnosis of patients with cerebral venous thrombosis [7]. Therefore, how to improve the early diagnosis level of thrombosis has become an urgent problem to be solved in the study of cerebral venous thrombosis.

**Fig. 1:**
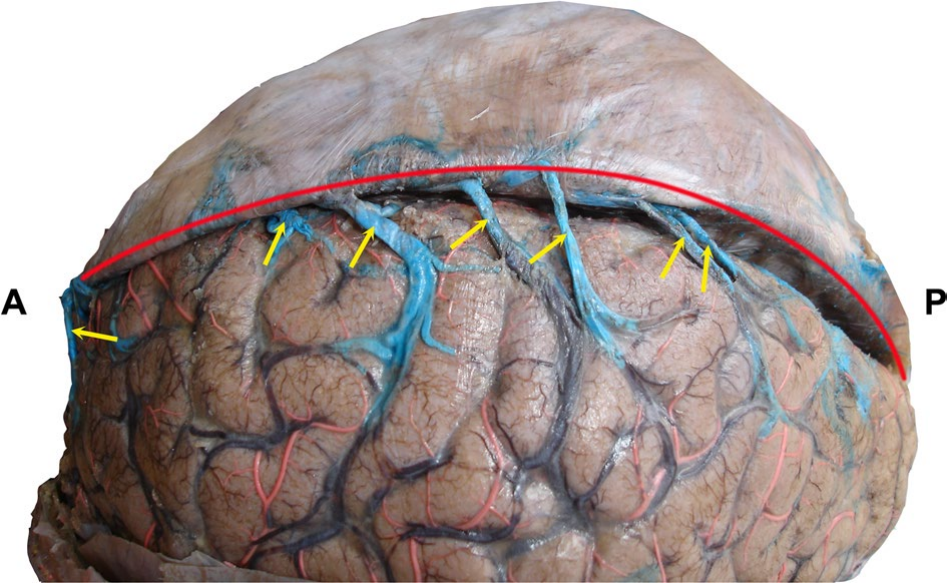
Anatomical picture of bridging veins (yellow arrow) entering the superior sagittal sinus (red line)

**Fig. 2:**
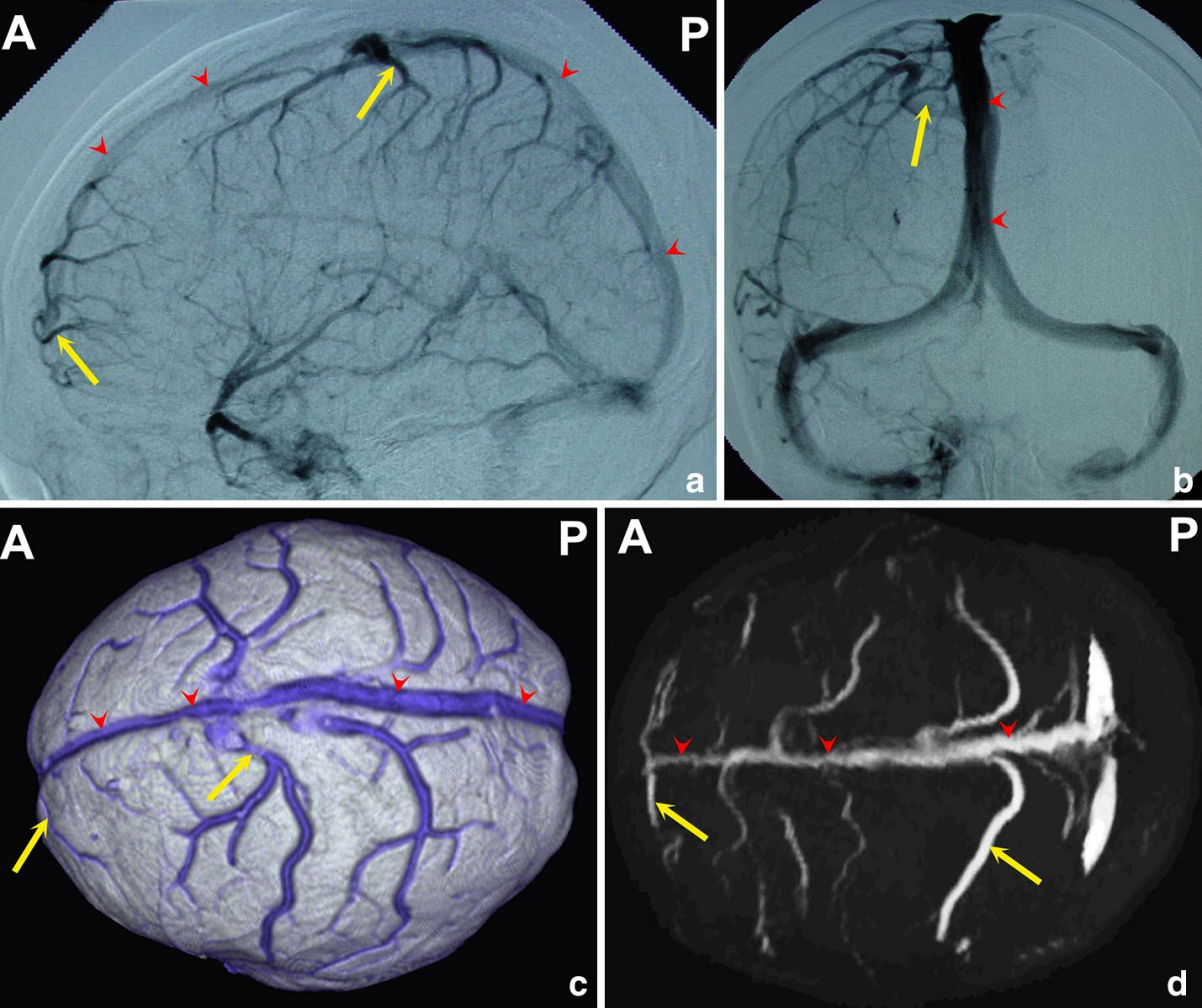
Bridging veins (yellow arrow) entering the superior sagittal sinus (red line) in lateral view (**a**) and anteroposterior view (**b**) of DSA, CTV (**c**) and MRV (**d**)

An international cooperation participated by 21 countries (including Portugal, Netherlands, France, and Mexico) shows that cerebral venous thrombosis is mainly in the superior sagittal sinus connected by the bridging veins [8], as illustrated in Figs. 1 and 2. However, the reason of its occurrence is not clear. In this study, we hypothesized that the cerebral bridging veins connecting superior sagittal sinus may have some specific morphological characteristics, then these parts of bridging veins and superior sagittal sinus are susceptible to the influence of pathogenic factors, which lead to the formation of thrombus.

The changes in hemodynamics such as wall shear stress (WSS) are an important factor for the formation of thrombus [9–11]. The WSS acts on vascular endothelial cells, and is parallel to the long axis of the vessel [12]. A certain level of WSS may have an effect of generating anticoagulant, inhibition of leukocyte adhesion and proliferation of smooth muscle [13–18]. The reference value of WSS in the arterial system is 1–7 Pa, while that in venous system is 0.1–0.6 Pa [19]. When the WSS is significantly lower than the normal value, the sharp reduction of the anticoagulant substance, enhancement of leukocyte adhesion and proliferation of smooth muscle can lead to thrombosis, atherosclerosis and other diseases [19, 20]. There is also some convincing research that compared with the low but steady WSS, sharp changes in WSS can easily lead to the occurrence of diseases [21, 22].

At present, computational fluid dynamics (CFD) is internationally used to simulate the movement of blood and other fluids. In the medical field, CFD has been widely used in the simulation of the occurrence and development of atherosclerosis, aortic dissection, aneurysm and other arterial diseases [23–29]; however, the hemodynamic simulation of the venous system has not been reported. Therefore, in this study, the hemodynamic physical models are established with the help of microanatomy observation data and CFD to determine the morphological features of thrombosis and find the predilection site of thrombus. Then, based on this, explanation of pathogenesis of cerebral venous thrombosis and imaging diagnosis are provided.

## Methods

### Micro-dissection

Six cases (12 sides) of formalin fixed adult cadaver brains provided by the Department of Anatomy in Anhui Medical University were chosen, each three cases for male and female, and the age was 42 ± 9 years old (34–59 years). After removing the calvaria by conventional craniotomy, the cavity congestion in superior sagittal sinus and internal jugular veins was flushed by intubations; then blue latex was injected into the superior sagittal sinus and internal jugular veins.

The dura mater was cut along 25 mm near superior sagittal sinus after 48 h, the adhesion between dura mater and arachnoid mater was carefully removed, and the bridging veins entering superior sagittal sinus were carefully separated. The bridging veins were found to be centrally located in the anterior and posterior segment of superior sagittal sinus. In accordance with the previous section standards [30], the bridging veins were divided into two groups: anterior group and posterior group. The diameter and angle of the bridging veins entering the superior sagittal sinus (entry angle) were measured.

### Computational fluid analysis

Models of one single cerebral bridging vein entering superior sagittal sinus were established from the anatomical data by CFD software ANSYS-Fluent. The inlet boundary conditions were entrance velocity. According to the measurement results of Chen et al. from the patients with selective craniotomy 1 year ago [31], the inlet velocity of superior sagittal sinus was 15 cm/s and the inlet velocity of bridging veins was 10 cm/s. The outlet boundary conditions was zero pressure. The wall is assumed to be smooth, and no slip condition is specified at the wall. The ambient pressure was the intracranial pressure of 1333 Pa, with fluid density of 1050 kg/m^3^ and viscosity of 4.24 × 10^−3^ Pa s (normal blood).

### Statistical treatment

The obtained data were processed by statistical software SPSS, and the results were expressed as $\bar{x} \pm {\text{s}}$ (min–max). The different results were compared by one-way ANOVA.

## Results

### Diameter and entry angle of the bridging veins

**Fig. 3:**
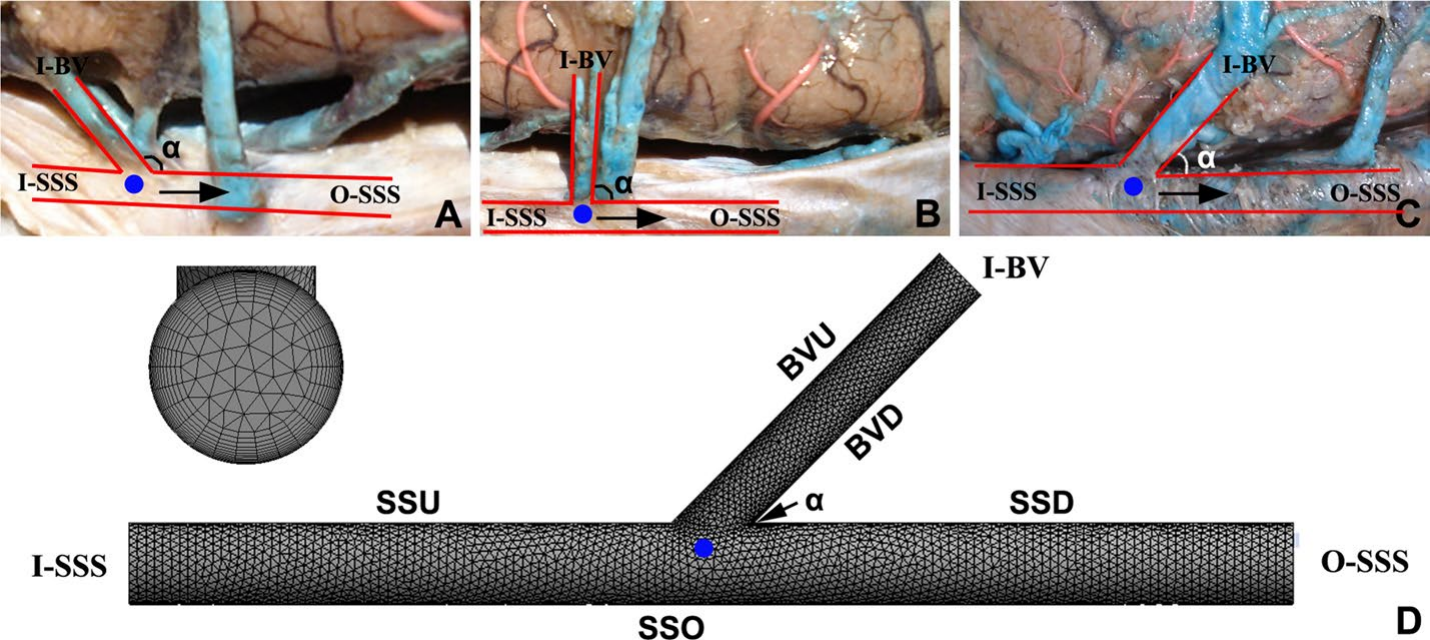
Establishment of the hemodynamic physical model. **A**–**C** The obtainment of the morphological data. The entry angle > 90° in **A** ≈ 90° in **B** and > 90° in **c**, respectively. **D** The grid after meshing and vessel boundary: *SSS* superior sagittal sinus; *BV* bridging vein; *Black circle* dural entrance which is the point that BV entering SSS; *α* entry angle which is the angle that BV entering the SSS; *I-SSS* inlet of SSS; *BV* inlet of BV; *O-SSS* outlet of SSS; *SSU* upstream wall of SSS from the dural entrance; *SSD* downstream wall of SSS from the dural entrance; *SSO* opposite wall of SSS from the dural entrance; *BVU* upstream wall of BV from the dural entrance; *BVD* downstream wall of BV from the dural entrance

A total of 137 bridging veins were observed; 62 of which entered the anterior segment of superior sagittal sinus (anterior group) with diameters of 2.0 ± 0.9 mm and entry angles of 93 ± 34°, while 75 of which entered the posterior segment of superior sagittal sinus (posterior group) with diameters of 3.0 ± 1.1 mm and entry angles of 43 ± 25°. Compared to the anterior segment of bridging veins, the diameters of posterior segment of bridging veins were enhanced, and the entry angles were obviously decreased (Figs. 1, 3, Table 1).

**Table 1:** Diameter and angle of bridging veins entering the superior sagittal sinus

Groups	Diameter (mm)	Angle (°)
Anterior group	2.0 ± 0.9 (0.6–5.2)	93 ± 34 (40–170)
Posterior group	3.0 ± 1.1 (0.8–5.8)**	43 ± 25 (10–90)**
Total	2.6 ± 1.1 (0.6–5.8)	66 ± 39 (10–170)

** Compared with bridging veins in anterior group, *P *< 0.01

### Stable value of wall shear stress in different vascular wall

According to the microsurgical anatomy data, 137 models of cerebral bridging veins entering the superior sagittal sinus were built, and the definition of the vessel walls of superior sagittal sinus and bridging vein is shown in Fig. 3D. Then, the WSS in a certain point is calculated as:

1 $$ \overline{{WSS_{{}} }} = \frac{{\iiint_{D} {\tau_{\omega } (x,y,z)d_{x} d_{y} d_{z} }}}{||D||}, $$

where *τ*_*w*_ is the WSS on the wall, and x, y and z are the 3D coordinates in space. *D* is the volume while *d* is the infinitisimal distance.

**Fig. 4:**
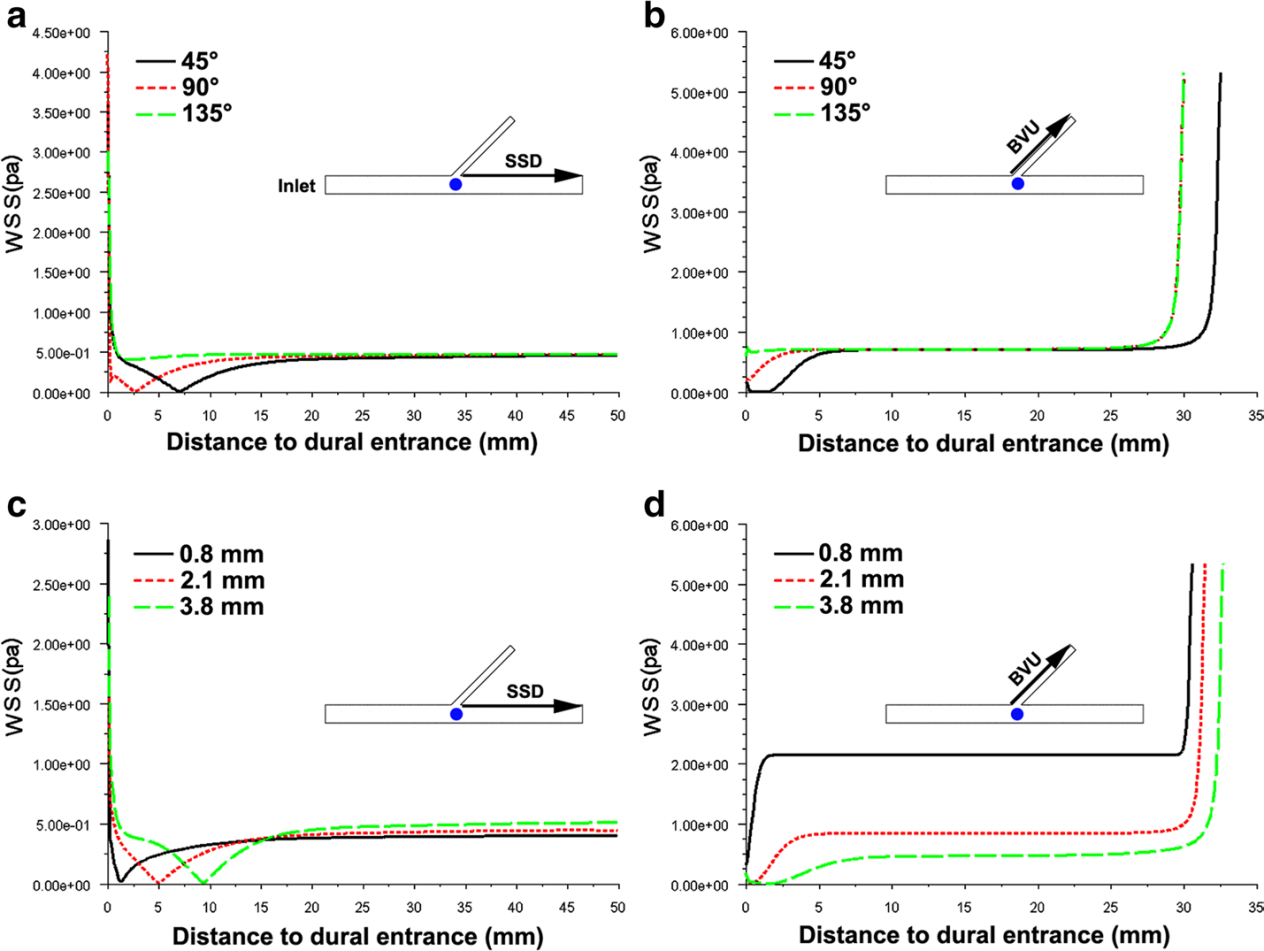
Line graphs of WSS in SSD and BVU under typical entry angle and diameter. **a**, **b** Typical entry angle of BV. **c**, **d** Typical diameter of BV. **a**, **c** WSS in downstream wall of SSS from the dural entrance (SSD). **b**, **d** WSS in upstream wall of BV from the dural entrance (BVU)

**Fig. 5:**
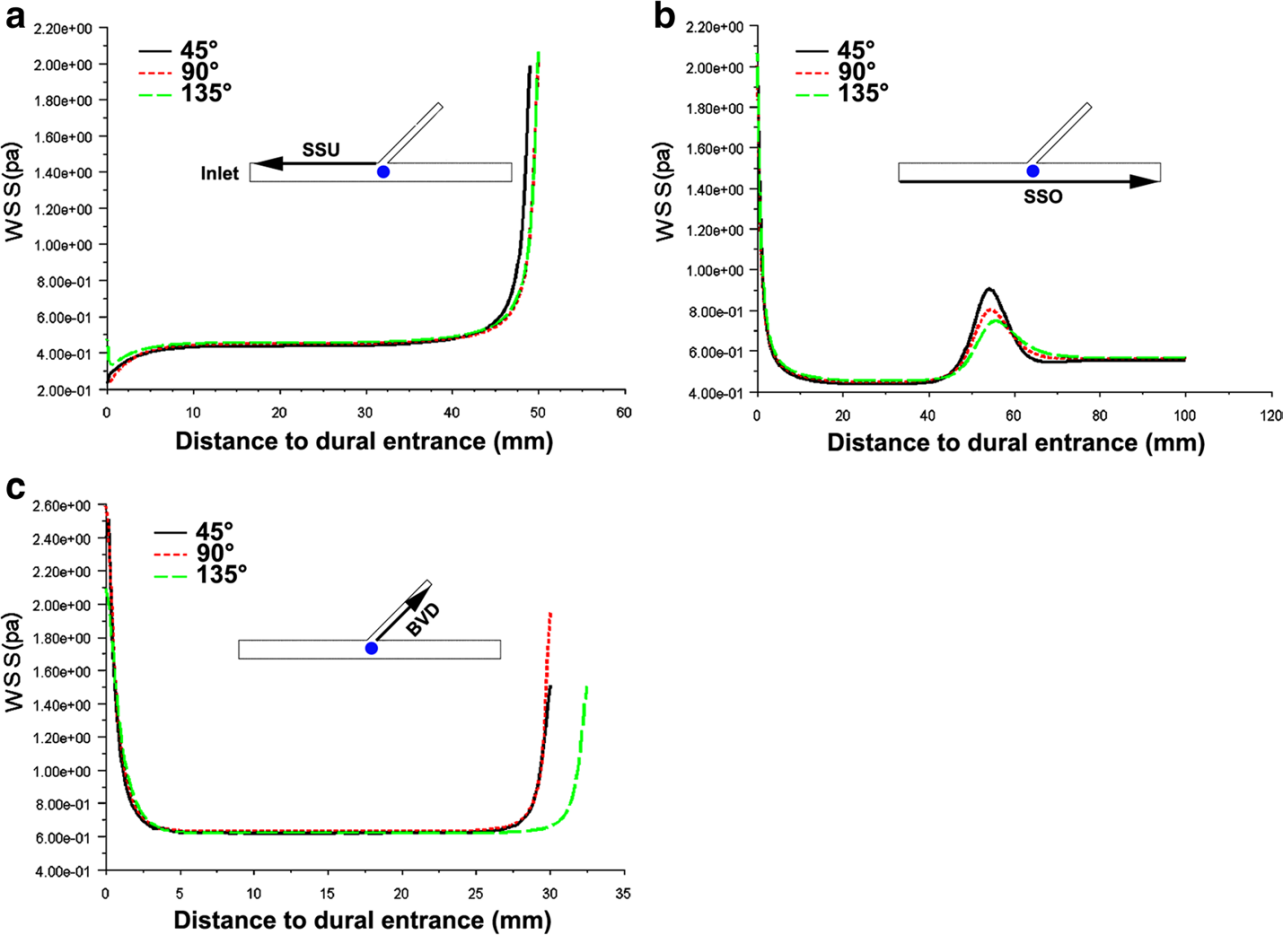
Line graphs of WSS in SSU, SSO and BVD. **a** WSS in upstream wall of SSS from the dural entrance (SSU). **b** WSS in opposite wall of SSS from the dural entrance (SSO). **c** WSS in downstream wall of BV from the dural entrance (BVD)

The WSS in all the vascular wall of cerebral superficial venous system were relatively stable, expect the inlets of vessel and the place near the entrance (Figs. 4, 5). As the WSS at opposite wall of superior sagittal sinus from the dural entrance (SSO) had significant different between the place before and after entrance (Fig. 5b), the SSO was divided into two segments: downstream of SSO (SSO-U) and upstream of SSO (SSO-D).

**Fig. 6:**
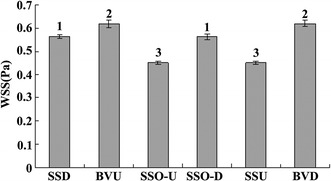
Stable value of WSS along the vessel wall in the cerebrovenous system. According to whether the WSS along different walls has statistical discrepancy, the walls of cerebrovenous system were divided into three groups: 1 SSD (downstream wall of SSS from the dural entrance) and SSO-D (opposite and downstream wall of SSS from the dural entrance), 2 BVU (upstream wall of BV from the dural entrance) and BVD (downstream wall of BV from the dural entrance), 3 SSU (upstream wall of SSS from the dural entrance) and SSO-U (opposite and upstream wall of SSS from the dural entrance)

The stable value of WSS in the whole cerebral superficial venous system was 0.544 + 0.072 Pa. According to the statistical difference, the stable value were divided into three groups: stable value in downstream wall of superior sagittal sinus from the dural entrance (SSD) and SSO-D was 0.563 + 0.009 Pa; stable value in upstream wall of bridging vein from the dural entrance (BVU) and downstream wall of bridging vein from the dural entrance (BVD) was 0.619 + 0.015 Pa; stable value in upstream wall of superior sagittal sinus from the dural entrance (SSU) and SSO-U was 0.450 + 0.007 Pa. The difference of WSS between groups was statistically significant, and there was no statistical significance in the group (Fig. 6).

### Comparison of wall shear stress between models with different entry angles and diameters

**Fig. 7:**
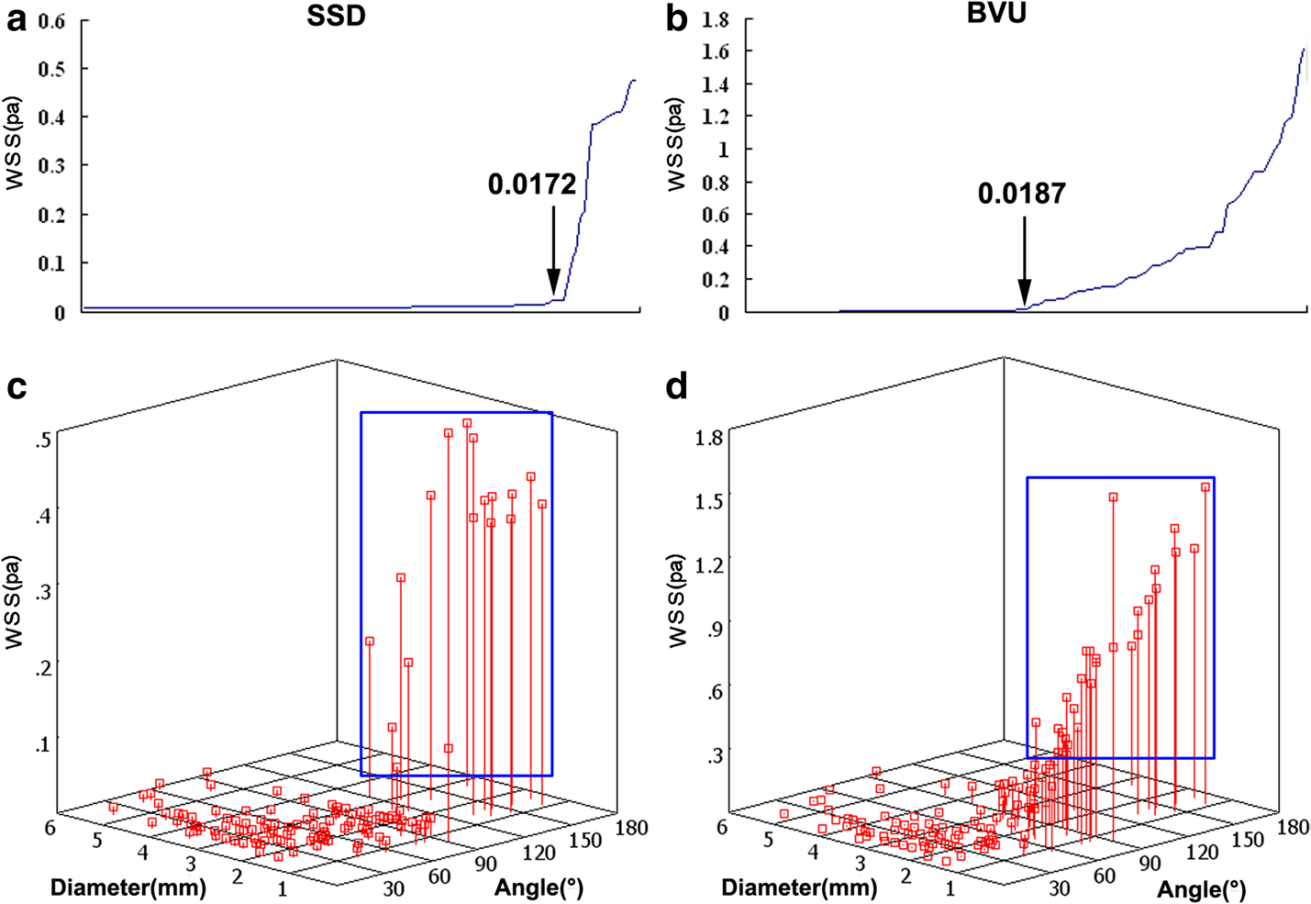
Minimum WSS in SSD and BVU. **a** The minimum WSS arrayed from low to high in SSD. **b** The minimum WSS arrayed from low to high in BVU. **c** The 3-D scatterplot of minimum WSS in SSD with various diameter and angles. **d** The 3-D scatterplot of minimum WSS in BVU with various diameter and angles

As shown in Fig. 4, when the BV entry angles are small and the diameters are large, the local WSS in the SSD and BVU were significantly decreased. In the other parts of the vessel wall, the differences of WSS among various models were not so obvious (Fig. 5). The minimum values of the above two WSS in SSD and BVU were arrayed from low to high, and are graphically displayed in Fig. 7a, b. It is seen that at the minimum value of around 0.017 Pa, there is a clear demarcation in the level of WSS.

Corresponding to the original data and the scatter plots as shown in Fig. 7c, d. In the SSD, it is seen that when the diameters of the bridging veins were less than or equal to 1.2 mm or the angles were larger than or equal to 105°, the WSS did not significantly decrease (and the minimum value of WSS was above 0.017 Pa). In the BVU, it is seen that when the diameters of bridging veins were less than or equal to 1.2 mm or the angles were larger than or equal to 65°, and the WSS did not significantly decrease.

According to the minimum value of the WSS in the scatter plots and graphs, in accordance with the different entry angles, the bridging veins models were divided into three groups: (10°, 65°), (65°, 105°)and (105°, 170°), as shown in Table 2. The data of bridging veins with diameters less than or equal to 1.2 mm were not included. It was observed that no matter how the entry angles changed, the WSS decreased significantly.

**Table 2:** The difference of minimum WSS in the models of BVs with various entry angles

Index	(10°, 65°)	(65°, 105°)	(105°, 170°)
SSD			
Minimum value (Pa)	0.008 ± 0.001 (0.007–0.010)	0.010 ± 0.001 (0.008–0.014)	0.338 ± 0.139 (0.065–0.477)**^##^
Position (mm)	9.4 ± 3.2 (4.4–17.0)	3.3 ± 1.8 (0.8–9.1)*	12.8 ± 17.4 (0.9–50.0)^##^
BVU			
Minimum value (Pa)	0.005 ± 0.002 (0.001–0.016)	0.189 ± 0.126 m(0.012–0.488)**	0.728 ± 0.296 (0.351–1.190)**^##^
Position (mm)	2.9 ± 2.5 (0.3–13.5)	0.2 ± 0.2 (0.0–1.1)**	2.1 ± 3.6 (0.0–10.5)^##^

*SSD* downstream wall of SSS; *BVU* upstream wall of BVs

* Compared with the models with the angle (10°, 60°), P < 0.05

** Compared with the models with the angle (10°, 60°), P < 0.01

^##^Compared with the models with the angle (60°, 100°), P < 0.01

The minimum WSS in SSD in each group were 0.008 ± 0.001, 0.010 ± 0.001 and 0.338 ± 0.139 Pa, respectively. The minimum value in the (100°, 170°) group was higher than those in the other two groups (P < 0.01); The minimum WSS in BVU in each group were 0.005 ± 0.002, 0.189 ± 0.126 and 0.728 ± 0.296 Pa, respectively. The differences between the three groups were statistical significant (P < 0.01). In the (10°, 60°) group, the minimum WSS in BVU was 63% of that in SSD. The differences were statistical significant (P < 0.01).

### Comparison of the wall shear stress in the anterior and posterior segments of bridging vein models

The bridging vein models were divided into anterior group and posterior group. As displayed in Table 3, in the anterior group, the minimum WSS in the SSD was 0.105 ± 0.164 Pa, at a distance of 5.6 + 9.2 mm from the dural entrance. The minimum WSS in BVU was 0.440 ± 0.426 Pa, at a distance of 0.7 ± 1.9 mm from the dural entrance. In the posterior group, the minimum WSS in SSD was 0.009 ± 0.001 Pa, at a distance of 9.0 ± 6.1 mm from the dural entrance. The minimum WSS in BVU was 0.043 ± 0.081 Pa, at a distance of 2.5 ± 2.6 mm from the dural entrance (Detailed data were shown in Additional file 1). Compared to the anterior group, the minimum value of the posterior vascular WSS was smaller, and the average distance from the dural entrance was longer.

**Table 3:** The differences of minimum WSS in anterior and posterior groups models

Index	Anterior group	Posterior groups	Total
SSD			
Minimum value (Pa)	0.105 ± 0.164 (0.007–0.477)	0.009 ± 0.001 (0.007–0.017)**	0.338 ± 0.139 (0.007–0.477)
Position (mm)	5.6 ± 9.2 (0.4–50.0)	9.0 ± 6.1 (1.4–48.7)*	7.4 ± 7.8 (0.4–50.0)
BVU			
Minimum value (Pa)	0.440 ± 0.426 (0.003–1.619)	0.043 ± 0.081 (0.001–0.357)**	0.223 ± 0.353 (0.001–1.619)
Position (mm)	0.7 ± 1.9 (0.0–10.5)	2.5 ± 2.6 (0.0–13.5)**	1.7 ± 2.5 (0.0–13.5)

Compared with the models of BVs entering anterior part of SSS, * P < 0.05, ** P < 0.01

## Discussion

The calculation processes of CFD are divided into five steps: geometric modeling, meshing, setting boundary conditions, solving and post processing. The geometry of the BV physical models, the dividing methods of meshing and the setting of different boundary conditions may influence the calculation results. The geometry of the BV physical models is considered to be the most critical factor to determine whether the results of the physical models were correct or not [32]. In this study, the geometry of the physical models was derived from the microsurgical anatomy photographs and data. This conforms to the reality, and it can help to obtain more accurate model analysis results.

The WSS is formed by friction between the blood flow and fixed vascular wall. A certain size and stable value of WSS may have an effect of generating anticoagulant, inhibition of leukocyte adhesion and proliferation of smooth muscle [20]. Due to the lack of relevant literature, it is difficult to determine the amount of WSS considered as abnormal in the venous system. The results of this study show that on the minimum WSS curves, the lowest WSS is 0.017 Pa, which is the most drastic change of the curve. Therefore, the WSS of less than 0.017 Pa is considered as a reference index to judge the abnormal WSS.

In this study, 137 models were established by using anatomical data, the WSS in the downstream wall of superior sagittal sinus from the dural entrance and the upstream wall of bridging vein from the dural entrance were significantly decreased. It can be seen from the scatter diagram (Fig. 4) of minimum WSS value, when the diameters of bridging veins were ≤ 1.2 mm, the minimum value of WSS was above 0.017 Pa, that is the WSS did not significantly decrease. When the diameters of bridging veins were ≤ 1.2 mm, no matter how the entry angle changed, the hemodynamics of superior sagittal sinus did not significantly change. Thus, the cerebral venous thrombosis is not easy to form when the bridging veins is ≤ 1.2 mm.

This study found that in the models of bridging vein diameters > 1.2 mm, the WSS decreased in the downstream wall of superior sagittal sinus from the dual entrance with the entry angle less than 105°, and the minimum WSS was under 0.014 Pa. When 65° < entry angle < 105°, the distance of minimum WSS from the dural entrance was 3.3 ± 1.8 mm. When entry angle < 65°, the average distance of minimum WSS from dural entrance was 9.4 ± 3.2 mm. It was obviously that the latter is greater than the former, that is, the reducing range was large. When the entry angles are smaller than 65°, the hear stress in the upstream wall of bridging vein from dural entrance was significantly decreased, and the minimum WSS was 0.005 ± 0.002 Pa. The reduction of WSS is an important factor for the formation of thrombus [20]. At the same time, the larger the range of WSS in an area, the more prone it is to thrombosis formation. Therefore, the harmful morphological characteristics of bridging veins were found to be: the entry angle of bridging veins injected into the superior sagittal sinus to be smaller than 65° and the diameter to be greater than 1.2 mm.

Previous studies have indicated that cerebral venous thrombosis usually occurs in the dural sinus and extends to bridging veins, while single bridging vein thrombosis is rarely seen [8]. Niggemann et al. have reported a case of a simple bridging vein thrombosis, and considered that cerebral venous thrombosis is more likely to occur in bridging veins [33]. The results of this study support this view. When the entry angle of bridging veins injected into the superior sagittal sinus is smaller than 65° and the diameter is greater than 1.2 mm, the minimum WSS in the downstream superior sagittal sinus wall is 0.008 Pa while that in the upstream is 0.005 Pa. Compared to the superior sagittal sinus wall, the WSS in the bridging vein wall reduces more obviously, and the tube wall is easier to be hurt. Therefore, thrombosis is more likely to occur in bridging veins than in the superior sagittal sinus.

The BV models were divided into two groups according to the different segments of bridging veins. Compared with the anterior segment group, the diameter of bridging veins in the posterior segment was larger, and entry angle of superior sagittal sinus was smaller. Bridging veins with large diameter and small entry angle may lead to the decrease of WSS. Compared with the anterior segment of bridging vein models, the minimum WSS in posterior group was smaller, and the distance from the dural entrance was larger. The distance from the minimum WSS to the dural entrance is 2.9 ± 2.5 (0.3–13.5) mm, while the lowest WSS is in the central position of the region where the WSS is reduced. The range of minimum WSS is about two times the distance from the dural entrance to the minimum WSS, which is 5.7 ± 5.1 (0.6–27.0) mm. As a result, the predilection site of thrombosis is on the upstream wall of cerebral bridging veins from the dural entrance, which is within 27 mm from the entrance.

The collateral circulation of bridging veins is abundant [34]. Due to the compensatory effect of adjacent veins, thrombotic occlusion of one or a few bridging veins usually does not cause obvious clinical symptoms. The superior sagittal sinus thrombosis causes backflow obstruction of all draining veins before the lesion location, and different measures of compensation. This leads to complications of cerebral hemorrhage, cerebral edema, venous infarction and so on, for which the treatment is relatively difficult [35]. The results of this study have shown that thrombosis is more likely to occur in bridging veins; when the disease process is accentuated, the disease can be gradually extended to the superior sagittal sinus.

## Conclusions

Our data suggest that the cerebral venous thrombosis occurs more easily when the diameter of a BV is greater than 1.2 mm and the entry angle is less than 65°. The embolus is formed earlier in the upstream wall of BVs in the posterior part of SSS. Therefore, in the early stages of the disease, the predilection site of thrombus in the image is observed carefully to enable early discovery of thrombus. Lesion migration to superior sagittal sinus can then be avoided by active treatments, which is of great significance for the prognosis of the disease and reduction of the incidence of complications.

## Additional file

**Additional file 1.** The detail data about the difference between the anterior and posterior segments of bridging vein models.

## Abbreviations

BV: bridging vein
BVD: downstream wall of BV from the dural entrance
BVU: upstream wall of bridging vein from the dural entrance
CFD: computational fluid dynamics
CTV: computed tomographic venography
DSA: digital subtraction angiography
MRV: magnetic resonance venography
SSS: superior sagittal sinus
SSU: upstream wall of SSS from the dural entrance
SSD: downstream wall of SSS from the dural entrance
SSO: opposite wall of SSS from the dural entrance
